# Male employment and female intra-household decision-making: a Mexican gold mining case study

**DOI:** 10.1007/s11150-020-09520-z

**Published:** 2020-11-18

**Authors:** Audrey Au Yong Lyn

**Affiliations:** 1 ETH Zurich, KOF Swiss Economic Institute, Leonhardstrasse 21, 8092 Zürich, Switzerland; 2 Munich Graduate School of Economics (MGSE), Ludwig-Maximilian University of Munich, Kaulbachstrasse 45, 80539 Munich, Germany

**Keywords:** Gold mining, Intra-household decision making, Global income shocks, Male employment, J12, D13, F62, L72

## Abstract

This study explores the effect of economic booms in male-dominated industries like mining on female intra-household decision-making power. Using the 2007–2008 global financial crisis as an exogenous event which led to a gold mining boom in Mexico, I find that women living in gold endowed municipalities experienced higher decision-making power contrary to some theoretical predictions. These results appear to be consistent with unitary household bargaining models which assume income pooling, as female decision-making power increased despite no changes in female labor force participation and an observed increase in male employment. Findings from a separate survey additionally show that while women residing in gold endowed states had higher decision-making power, they were also more likely to suffer from intimate partner violence (IPV). This suggests that a woman’s intra-household decision-making authority is not necessarily negatively correlated with her risk of IPV as posited in feminist theory.

## Introduction

Mexico has a long-standing history of gold mining, where the practice of mining gold dates back to the pre-Hispanic times and contributed greatly to Latin America’s economic expansion during the colonial era. It was not until the last two decades however, that the Mexican gold mining industry took off. Prior to the 2000s, Mexico predominantly focused on silver production, as the country had traditionally been the number one producer of the world’s silver. Due to market speculation of the impending 2007–2008 financial crisis, global gold prices started increasing in 2003 and spiked sharply between 2006 and 2011, with Mexican gold production following the same trend. This event, together with the influx of foreign direct investments (FDI) for mining explorations, inevitably generated a shift in focus from silver to gold mining in Mexico during this time period (Secretaría de Economía, 2013).^1^ According to the National Institute for Statistics and Geography (INEGI), the production of gold in Mexico increased three-fold at an average rate of about 5.3% each year between 2000 and 2011. Relative to the world however, Mexico’s global share in gold production during the mining boom only increased from about 0.3% in 2000 to 2.8% in 2011 (The Observatory of Economic Complexity (OEC)). Notwithstanding, Mexico’s rich endowment in precious metals like gold in addition to the gold mining boom due to the 2007–2008 global financial crisis, provides an ideal setting for studies that aim to understand how male employment stimulated by mineral-led activities, impact female intra-household welfare outcomes like decision-making and intimate partner violence (IPV).

For many centuries, superstition has kept Latin American women away from mining as it was believed that if a woman went near a mine, it would become jealous, hide its wealth and cause catastrophes (Arcos et al. 2018). Lutz-Ley and Beuchler (2020) additionally note that because mining takes place in remote areas, miners are subject to long journeys, uncomfortable settings, and risky conditions. For these reasons, the mining industry has typically been male-dominated. In Mexico, despite efforts to increase female participation in the mining sector, the proportion of women in mining only increased from about 6% in 2004 to 13% in 2009, and subsequently decreased to 11% in 2014 (National Institute for Statistics and Geography (INEGI), 2020).^2^ Figure 4 of the appendix also plots the absolute number of employees in gold mining by gender, between 1994 and 2014. The graph shows that while male employment in gold mining increased sharply especially between 2009 and 2014, female employment in gold mining only rose marginally during the same time period. Subsequently, in a mining report by the African Union (2009), it was suggested that booms in such male-dominated sectors would result in a decrease in women’s intra-household decision-making power. The underlying logic is that as the mining sector flourishes and more men than women gain employment, women become increasingly reliant on their partners’ incomes. Alternatively, busts in the mining industry could also decrease women’s decision-making power through poorer employment prospects related to industrial crowding out effects. In a study on the U.K. by Aragon et al. (2018), coal mine closures were found to be associated with an increase (decrease) in male (female) employment due to the crowding out of female-dominated sectors like manufacturing. In the context of oil extraction in the U.S. however, Maurer and Potlogea (2020) did not observe any gender-biased crowding out effects, contrary to Aragon et al. (2018) findings. Ultimately, the impact of changes in extractive industries (EI) on female employment and in turn, intra-household decision-making outcomes remains inconclusive and renders further investigation.

To date, only one paper by Tolonen (2018), has explicitly explored the relationship between mining and spousal decision-making dynamics. In a cross-country study on Sub-Saharan Africa, the author found no effect of local gold mine openings on women’s intra-household bargaining outcomes. This in turn raises the question of whether the commonly assumed negative relationship between gold mining and intra-household female decision-making power is consistent with real world data. This paper therefore endeavors to further test the hypothesized negative relationship between booms in male-dominated industries like mining, and female decision-making power in a Latin American country like Mexico. From a cultural and socio-economic perspective, the Sub-Sahara African region and Latin America are highly distinct. The impact on female decision-making outcomes generated by a mining boom could therefore be different across geographic regions.^3^ Tolonen’s (2018) findings from her cross-country study on Sub-Saharan Africa may thus not be generalizable to countries like Mexico in the Latin American region. In addition, since more indigenous women in Mexico have been documented to live in mining communities and are simultaneously poorer and subject to traditional gender stereotypes, understanding the impact of economic booms in male-dominated sectors like mining is important for enhancing and facilitating gender equality efforts among these particular demographic groups (Lutz-Ley and Buechler 2020).

This paper contributes to existing literature in additional ways. First, no previous studies to my knowledge have used exogenous movements in global commodity prices to evaluate the effect of a mining boom on female intra-household decision-making power and intimate partner violence (IPV). In particular, this study exploits the sharp rise in world gold prices between 2003 and 2011, as well as the differences in gold endowment across municipalities and states in Mexico as sources of variation. The paper also utilizes two different data sources, one at the municipality level (*MxFLS*) and the other at the state level (*ENDIREH*), to identify changes in not only female bargaining power, but also IPV outcomes during the gold mining boom respectively. To date, only two studies on Sub-Saharan Africa have analysed the impact of mining on IPV. In a cross-country analysis on Sub-Saharan Africa, Kotsadam et al. (2016) found no significant relationship between both factors, whereas in a more location-specific study on Eastern Democratic Republic of Congo (DRC), Rustad et al. (2016) discovered that women who lived closer to artisanal and small-scale mining were more likely to experience sexual violence. In this study on Mexico, due to the detailed information on IPV provided by a state-level (*ENDIREH*) data set, I am able to segregate IPV into four different forms (physical, sexual and emotional abuse and threats of violence), unlike previous studies by Kotsadam et al. (2016) and Rustad et al. (2016) that have only examined harder types of IPV like physical and sexual violence.

The results from the analyses show that women living in gold endowed states were more likely to suffer from various forms of IPV, though the types of IPV experienced by women were different for those who were poorer and wealthier. Given the spike in domestic violence during COVID-19, this study is therefore particularly relevant as it helps to shed some light on how changes in male employment as a result of economic booms or busts (in the context of COVID-19) affect women’s risk of IPV, especially in low-middle income countries like Mexico. The findings also reveal that women residing in gold endowed municipalities experienced an increase in their decision-making power at home, which was likely to be driven by a rise in male employment probabilities. Contrary to non-unitary household bargaining models (see McElroy and Horney 1981, Lundberg and Pollak 1994 and Lommerud, 2003) that predict a decline in a woman’s household bargaining power along with a concurrent increase in male employment opportunities generated by a mining boom for instance, the results from this study suggest that an increase in a husband’s outside option through better employment prospects relative to his wife’s, may not necessarily hamper her intra-household decision-making power ability. This finding can be juxtaposed against unitary household bargaining models that predict the pooling of household income (Samuelson 1956). From a policy standpoint, it subsequently elucidates how income resources are distributed within the household, which carries important implications for the effectiveness of cash-transfer programs in reducing IPV for example.

The rest of the paper is organized as follows: section II provides a background on gold mining in Mexico and discusses relevant theories relating income-generating opportunities to intra-household female decision-making; section III describes the data and empirical method used in this study; section IV presents the main results of the paper, discusses possible channels and additional outcomes like IPV, and reports a set of robustness checks; section V finally concludes.

## Background and existing literature

### Gold Mining in Mexico

**Fig. 1:**
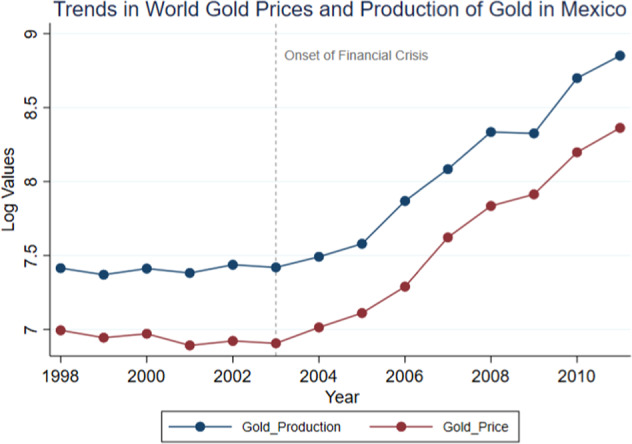
Trends in world gold prices and gold production in Mexico. *Notes*: This figure plots trends in global gold prices and the production of gold in Mexico. Each point is the logged value of average prices over each year and the total production in each year. The blue (red) line denotes production (price). Data on Mexico’s gold production come from the National Institute for Statistics and Geography (INEGI), and data on world gold prices is from the London Metal Exchange’s (LME) monthly historical price series

This study specifically focuses on gold mining in Mexico. Due to market speculation of the 2007–2008 global financial crisis, the global demand for gold began increasing in the few years leading up to the crisis in 2003. Figure 4 of the appendix supports this phenomenon, and shows an increased demand for labor in the gold mining sector particularly for men, between 2004 and 2014. By 2011, the price of gold was approximately six times higher than pre-shock levels in 2002, with the steepest increase occurring between the end of 2006 and 2011.^4^ This event subsequently stimulated a gold mining boom across many mineral-led countries like Mexico, as gold production intensified in response to the spike in global gold prices (Engineering and Mining Journal 2011). Figure 1 shows the natural log of the prices and production of gold through these shocks between 1998 and 2011.

As can be seen, global gold prices increased steadily from 2003 to 2011, and gold production in Mexico followed the same trend. It is worth noting that although the prices of other precious metals like silver rose as well, the increase was not as substantial as compared to gold. While the prices and production of silver nearly doubled between 2009 and 2011, they were relatively constant before that period (see Figure 5 in the appendix). Moreover, silver has a much lower value to weight ratio than gold, where the gold-silver price ratio was 84:1 at the depth of the global financial crisis.^5^ Therefore despite Mexico being a large producer and exporter of silver, there is little temporal variation to exploit in silver mining production for this study. Accordingly, I test if the production of gold in Mexico responded to world gold prices, by conducting a first-stage analysis of the relationship between the two variables. The *t-*statistics of the effect of current prices on future production 1,2 and 3 months after (*t*+1 to *t*+3) are highly statistically significant (18.09, 20.98, 20.96 respectively), indicating that local production responded strongly to global prices.^6^ Figure 1 additionally shows that local gold production and world prices followed highly similar trends during the sample period.

**Fig. 2:**
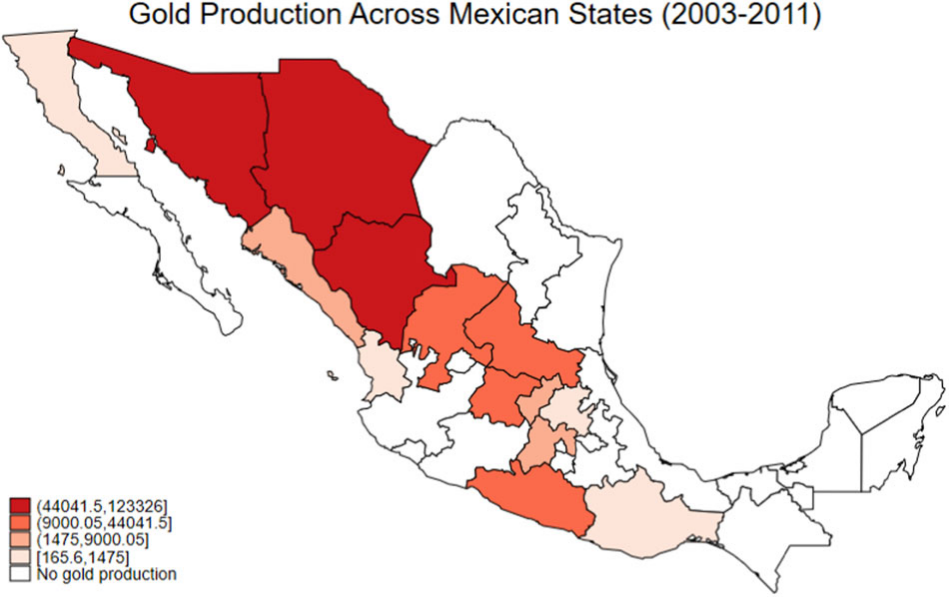
A graphical representation of gold production across Mexican states between 2003 and 2011. *Notes*: The graph shows the total production of gold (in kilograms) in each state between 2003–2011. The darker the shaded area, the greater the quantity of gold produced in the state. Data on gold production come from the National Institute for Statistics and Geography (INEGI): Banco de Información Económica (BIE) series

Most of the gold mining in Mexico is conducted by large-scale companies located in the north and central regions. Figure 2 illustrates the geographical variation in gold production across the respective Mexican states between 2003 and 2011 specifically. The graph shows that three northern states: Sonora, Chihuahua and Durango contribute to the largest shares of gold produced in the country. While there is some gold mining activity in south-eastern states like Guerrero and Oaxaca, the average volume of gold produced in the north is approximately 4.5 times more than in these states. In total, about 14 out of 32 Mexican states are actively involved in the mining and production of gold, albeit the remaining 18 states also do produce gold but in much smaller quantities, contributing to only 0.4% of the total gold produced in Mexico.

Typically, the mining of gold is done through open-pit operations that use capital intensive technology, and many gold mining businesses in Mexico are owned by large international corporations or businesses rather than by locals.^7^ Much of the Mexican soil is also favorable for the practice of mining exploration and exploitation, where approximately two-thirds of the national territory contain mineral resources that are suitable for mining purposes (Hurtado and Salazar 1999). On average, it is estimated that exploration activities of a mining area take 10 years, and an additional 20 to 30 years for exploitation (The Mexican Geological Service (SGM) 2020).^8^ In the Mexican context, the relationship between *ejidos* and miners has also played a major role in mining production over the last three decades. *Ejidos* are essentially areas of land that are used for collective agricultural practices, whereby community members are assigned allotments on which they farm on, and have joint ownership over the land with other community members. In 1993, the mining law reform allowed *ejidos* and properties of agrarian communities to be privatized, meaning that in order for miners to carry out exploitation work on *ejidos*, they would have to have an agreement with the owners of the land, and have it registered with the agrarian registry (Penman 2016). Under these circumstances, if a consensus between the owner of an *ejido* and prospective mining business was not reached, miners would not be able to conduct exploitation activities.^9^

### Mining, employment and female decision-making power: conceptual framework

#### Non-unitary household bargaining models

Non-unitary household bargaining models in economics predict that external shift parameters such as an increase in female job opportunities for instance, are key determinants of a woman’s bargaining power.^10^ These external environmental factors are typically termed as the ‘outside option’, and are a function of an individual’s decision making authority (McElroy and Horney 1981; Lundberg and Pollak 1994; Lommerud 2003). Subsequently, the theory suggests that a change in a woman’s outside option is sufficient to alter her aggregate bargaining power within the household. Improvements in relative or absolute job opportunities for women due to gold mining for example, should thus increase female bargaining power through the outside option. In regions like Sub-Saharan Africa, Kotsadam and Tolonen (2016) found that the intensification of mining activities generated more jobs for women in complementary sectors like the service industry. However, in a subsequent study on Sub-Saharan Africa by Tolonen (2018), the author found no impact of gold mining on spousal decision-making power, indicating that an improvement in a woman’s outside option through an increase in job opportunities may not necessarily guarantee an increase in her intra-household bargaining power. Whether the Mexican gold mining boom affected female decision-making power through employment, therefore remains an empirical question.

The extent to which an improvement in a woman’s outside option can be leveraged to her advantage also depends on how male income or employment changes relative to females’ during a gold mining boom. The prediction of non-unitary household bargaining models is that if relative male income or employment increases, the intra-household gender wage gap widens and in turn lowers women’s decision-making power. In several studies on the Middle East and the U.K, booms in industries like crude oil and coal decreased female labor force participation due to higher male wages and increased household wealth, which subsequently crowded out female-dominated sectors like manufacturing (Ross 2008; Aragon et al. 2018). Considering that mining is still a male-dominated activity, economic booms in the sector in theory, could disproportionately increase employment prospects for men and decrease female intra-household decision-making power (African Union 2009).

### Unitary Household Bargaining Models

Unitary household bargaining models on the other hand, assume the pooling of household income which implies that the household demand for goods and leisure are not dependent on the sources of income (i.e. the individual income contribution of the wife and the husband to household wealth), but rather the aggregate household income. Samuelson (1956) suggested that as long as there is a general consensus among household members on the aggregation of household income and preferences, the income pooling assumption is satisfied. Though the unitary model has been widely criticized for its over-simplicity and lack of applicability to real world data (see Lundberg and Pollak 1994; Browning et al. 2006; Bobonis 2009; Cherchye et al. 2012), there is evidence that some developing countries like India, still follow the unitary framework (see Neff et al. 2012; Klasen and Pieters, 2015). Consequently, under the unitary household bargaining model, a woman’s intra-household decision-making authority is dependent on changes in the total household wealth, rather than on changes in her absolute or relative income prospects. In this regard, the outside option characterized by better employment opportunities in gold mining for instance, will not affect her intra-household decision-making power over the consumption of goods and services, unlike what is predicted by non-unitary household bargaining models. A total increase in household finances is therefore sufficient to increase her intra-household decision-making power over consumption goods and services.

## Data and empirical method

### Data

#### Dependent variables

This study draws from a panel data set to elicit the effect of the gold mining boom on female decision-making power in Mexico. Specifically, it utilizes the Mexican Family Life Survey (MxFLS), which fortuitously overlaps with the timing of the gold mining boom as a result of the 2007–2008 global financial crisis. The *MxFLS* is a nationally representative panel survey containing information on the decision-making power of married or cohabiting couples, and interviewed approximately 8400 households and 35,000 individuals in more than 150 municipalities across Mexico.^11^ It also boasts low attrition rates with virtually 90% of households re-interviewed and tracked across all three survey waves.

To capture the differences in women’s intra-household decision-making power before and after the spike in gold mining production, I use all three waves of the *MxFLS* conducted in 2002, 2005–2006 and 2009–2010. In particular, I utilize the first wave of the MxFLS (2002) as the pre-treatment sample, and the last two waves (2005–2006, and 2009–2010) as the post-treatment sample given that gold prices only started increasing from 2003 onwards (see Fig. 1). Decision-making questionnaires specifically ask couples within a household about who made the decisions over twelve types of goods and services (children’s goods, private goods, spouse’s goods and household expenditures).^12^ Accordingly, I construct an average decision-making power index over all twelve decision-making questions, where each decision-making question is normalized with a mean of 0 and standard deviation of 1.^13^ The *MxFLS* additionally provides detailed information about an individual’s indigenous status, education, age, employment, month and year of interview, perception of safety, the number of kids at home, indicators of community and transport network quality, and most importantly, it notes the presence of a woman’s spouse during an interview which is included as a control to mitigate measurement errors resulting from possible reporting biases.^14^

#### Explanatory variables

In order to examine the effect of the gold mining boom on female decision-making power outcomes, I use exogenous global commodity shocks characterized by movements in gold prices. Considering that local prices of gold are likely to be endogenous to female intra-household bargaining power outcomes, for instance, mine openings or exploitation activities, could have been influenced by local gold prices, I use world gold prices instead, which is taken from the *United States Geological Survey (USGS)* historical statistics for mineral and material commodities. In this context, the exogeneity assumption holds reasonably as world gold prices are unlikely to be influenced by women’s intra-household decision-making behavior in Mexico. Moreover, during the sample time frame, Mexico was not a major global exporter and producer of gold. Between 2002–2011, Mexico contributed to only 1.63 and 1.66% of the world’s total gold exports and production respectively (The Observatory of Economic Complexity (OEC) 2019). Given the country’s small open gold economy, the issue of endogenous global gold prices is thus less of a concern due to Mexico’s insignificant share of global gold production and exports, which arguably has a negligible effect on world gold prices.

Next, since the drug war initiated by President Felipe Calderón in 2007 reportedly encouraged drug cartels to switch from trafficking to gold mining, it is not possible to rule out that gold mining projects during the sample time frame were influenced by levels of violence, which in turn could have impacted female bargaining power outcomes (Global Initiative against Transnational Organized Crime (GIATOC) 2016). To circumvent this potential endogeneity, I use historical mining concessions data from 1973 to 1983 as a proxy for gold mineral endowment.^15^ In essence, mining concessions are land areas that are allocated and permitted by the government for the extraction of minerals such as gold.^16^ The data contains information on the land area granted for the exploitation of gold, and is taken from *Mexican Ministry of Economy* which provides information on the intended gold mining land area (in hectares). Location information such as the municipality, and the date during which the gold mining concession was granted is also provided. I omit concessions that listed the mining of other minerals such as copper and lead, among other minerals, to elicit and isolate changes in gold mining activities between 2003–2011, the period during which gold prices started increasing. Two states: Mexico City (‘Federal District of Mexico’) and Quintana Roo, are also excluded from the analysis, as information on historical mining concessions for these states are not available.^17^ Summary statistics of dependent and independent variables are presented in Tables 7 and 8 of the Appendix.

#### Additional data sources

In the baseline regressions, I include the homicide rate per 100,000 people as a control to account for the Mexican drug war that overlapped with the gold mining boom. Data on homicides is drawn from the death registry of the *Vital Statistics* edition provided by the National Institute for Statistics and Geography (INEGI). Public death certificates are released by the INEGI annually, and contain details on the cause of death, the day, month and year of death as well as the municipality and state in which the death occurred for the years 1990–2011. The availability of this data permits the selection and inclusion of only intentional homicides, and the matching of homicide rates to individuals’ decision-making outcomes months prior to their *MxFLS* interview. The *National Agrarian Registry (RAN)* also provides information on the number of *ejidos* per municipality and state which is added as a heterogeneous market trend in baseline regressions to control for initial municipality conditions.

As a robustness test, I draw from an alternative data set, the Mexican National Survey on the Dynamics of Household Relations (ENDIREH), to cross-check female decision-making power outcomes observed in the *MxFLS*. *ENDIREH* is a nationally representative repeated cross-sectional survey containing information on women’s intra-household decision-making outcomes and self-reported measures of IPV, which is used as an additional outcome in the paper. Like the *MxFLS*, the survey overlaps with the timing of the gold mining boom, and was conducted in 2003, 2006 and 2011. Altogether, *ENDIREH* surveyed 34,184, 133,398 and 152,636 women who cohabitated with a partner or husband, and who were 15 years and above in 2003, 2006 and 2011 respectively.^18^ In particular, women were asked if they experienced any form of IPV in the 12 months prior to the interview as well as their decision-making power over various sets of goods and services. It is important to use *ENDIREH* to examine the effects of the gold mining boom on intra-household female welfare outcomes related to domestic violence for instance, as the *ENDIREH* is currently the only available data set in Mexico that contains detailed information about women’s IPV experiences. Different from the *MxFLS* however, location data for the *ENDIREH* is only available on the state rather than municipality level, as the survey was conducted to get a general gauge of the intensity and severity of domestic abuse across Mexico.

### Empirical strategy

An important assumption is that the production of gold which essentially serves as the treatment variable, intensified during the onset of the global financial crisis in 2003. Since the goal is to examine how intra-household female decision-making authority was influenced by changes in employment due to the gold mining boom over time, it is necessary to ensure that there is variation in gold mining production activities stimulated by the global economic downturn. In order to test this assumption, I run a first-stage regression analysis of the relationship between world gold prices and Mexican gold production. As previously discussed in section II.1, the association between the two variables is highly significant and positive. Figure 1 provides further support for this assumption which shows that world gold prices and gold production in Mexico exhibited similar trends. The availability of data on the month and year of interview in the *MxFLS* additionally allows the matching of individuals to monthly values of world gold prices.

One of the limitations of this study however, is the paucity of geocoordinate data in both the *MxFLS* and *ENDIREH*. As such, calculating the exact distance of individuals to mines is not possible, unlike studies on Sub-Saharan Africa by Kotsadam and Tolonen (2016) and Kotsadam et al. (2016) for instance, that use the Demographic Health Survey (DHS) series containing geocoordinate data. Moreover, due to limited data availability on historical gold mining projects, I utilize historical gold mining concessions as an alternative, which is a continuous endowment variable indicating the land area designated for the exploitation and extraction of gold. Municipalities that have larger mining concession areas therefore signify greater gold endowment compared to those with smaller mining concession land areas. In this respect, the treatment intensity is determined by the size of gold mining concession areas in the municipalities where women reside. It is also worth noting that since concessioned land areas may be explored but not eventually exploited, the empirical strategy follows an intent-to-treat approach which relies on the assumption that rising gold prices encouraged the exploitation and production of gold. Lastly, out of the 136 municipalities represented in this study, approximately 39% have land areas designated for the mining of gold, ranging from 18 to 3362 hectares (18,000 to 33,622,813 square meters).

In order to fully exploit changes in gold mining production activities due to the global economic crisis, I interact historical gold mining concession areas with the global gold prices. Doing so should help to elicit the differences in employment and intra-household bargaining outcomes between women who live in municipalities endowed with gold during the mining boom, and those who do not. On this account, the empirical strategy follows a difference-in-differences method of estimation, and capitalizes on the temporal and geographical variation in gold mining production across Mexico. Accordingly, the baseline regression takes the following form:

1 $$Y_{i,m,t} = B_0 + B_1\left( {Gendowment_m \ast Price_{t - 5}} \right) + B_2X_{i,t}^\prime + B_3Z_{m,t}^\prime + \emptyset _t + \pi _i + \varepsilon _{i,m,t}$$

Where *Y*_*i,m,t*_ is the average decision-making power index of woman *i* in municipality *m* at time *t*, *Gendowment*_*m*_ is the log of the land area claimed by gold mining concessions between 1973 and 1983 in municipality *m*, *Price*_*t*−5_ is the *average* world price of gold 5 months prior to the individuals’ interview month, $X_{i,t}^\prime$ and $Z_{m,t}^\prime$ are vectors of individual- and municipality-level covariates such as woman’s age, age squared, indigenous status, education level, number of children, the presence of her spouse during the interview, the homicide rate per 100,000 inhabitants, the introduction of domestic violence and divorce laws, and time trends interacted with municipal characteristics such as rural-urban status, transport network quality, community quality and the presence of *ejidos*, to account for heterogeneous market trends that may be correlated with initial municipal conditions.^19^ ∅_*t*_ and *π*_*i*_ are month and individual fixed effects respectively, and *ε*_*i,m,t*_ is the usual disturbance term.

It is crucial to control for municipality homicide rates as higher homicide rates have been found to lower women’s bargaining power at home (see Tsaneva et al. 2018).^20^ Due to the drug war, the Mexican mining industry grew in its susceptibility to drug-related criminal activity as the rising gold prices and concurrently declining profitability of drug trafficking from Mexico to the United States stimulated drug cartels to switch from trafficking to gold mining (Global Initiative against Transnational Organized Crime (GIATOC) 2016). Additionally, from the mid-1990s to the mid 2000s, Mexico implemented various divorce laws and reforms that criminalized domestic violence. Details on the dates of these various reforms can be found in Beleche’s (2017) study which examines the effect of these laws on female suicide in Mexico. Given the evidence that divorce law reforms and domestic violence laws improve a woman’s outside option, and hence her intra-household decision-making power (see Chiappori et al. 2002; Rangel 2006; Stevenson and Wolfers 2006), it is important to include the timing of these reforms as controls in the benchmark regression. Lastly, while the lag of the average gold prices in the last 1, 2, 3, 4, and 6 months also generates significant estimates for female decision-making power outcomes, this paper uses the average prices of gold over the last 5 months prior to the interview month of the individual, as this mean composite measure produces the most pronounced effect of the mining boom on women’s intra-household bargaining power. Because gold mining production might take some time to respond to changes in prices and hence generate lags in production and hiring adjustments, and since individuals may not immediately respond to economic stimulus, the more prominent results produced by the average of gold prices over the last 5 months is conceivable. In Fig. 7 of the appendix, I additionally present a coefficient plot of a woman’s mean decision-making power index according to the average gold prices over the last 1–6 months. In summary, the figure provides evidence that the choice of using average gold prices over the last 5 months is not arbitrary (the coefficient plot is a quadratic function with a maximum point at the 5^th^ lag).

Next, the inclusion of individual fixed effects is also especially advantageous given the nature of this study as individual decision-making behavior for instance, could vary greatly across individuals due to latent individual traits. Month of interview fixed effects additionally account for time trends that vary uniformly across municipalities. A potential threat to identification remains however, due to selective migration. For instance, women and men in the *MxFLS* could have moved to mining municipalities across the three survey waves in search of better job opportunities. It could also be that individuals migrated away from municipalities that were more dangerous as a result of the drug war, which has been reportedly associated with gold mining. Intra-household female welfare estimates could therefore be either upward or downward biased, depending on each specific scenario. Albeit an examination of the *MxFLS* shows that only 2.8% of women changed their municipalities of residence across all three survey waves, I nonetheless fix individuals to their municipality of residence in the first survey wave (2002) regardless of if they relocated to other municipalities over the years, to rule out any biases arising from selective migration. Consequently, the *MxFLS* results represent the intent-to-treat (ITT) effect as opposed to the average treatment effect (ATE) of the relationship between the gold mining boom and women’s intra-household decision-making power.

## Results

### The effect of the gold mining boom on women’s decision-making power

Table 1 show the results from estimating Eq. (1) which estimates the effect of the gold mining boom on women’s mean overall decision-making power and their decision-making authority over individual goods and services. Specification (1) includes individual and month fixed effects but no controls, and (2) adds baseline controls and non-parametric trends interacted with municipal characteristics aforementioned in section III.b, to account for heterogeneous market trends correlated with initial municipality conditions. Taken together, the results show that contrary to the commonly hypothesized negative relationship between mining and intra-household female decision-making power, women in gold endowed municipalities experienced an overall increase in their ability to partake in household decision-making processes. A more disaggregated analysis of the different categories of goods and services shows that the improvement in their average aggregate bargaining power was driven by three specific types of goods: food expenditures, money given to their own parents, and contraceptive use. The findings for the remaining categories of goods and services are presented in Table 9 of the appendix, and do not show a significant relationship with the gold mining boom.

**Table 1:** The effect of the gold mining boom on female decision-making power (*MxFLS* data)

	(1)	(2)	Mean (std. dev.)
Mean DMP (all goods)	0.001 (0.002)	0.006*** (0.002)	0.541
Observations (*N*)	10,462	9123	
*DMP (individual goods):*			
Food expenditures	0.014** (0.006)	0.018*** (0.006)	0.829
Observations (*N*)	7075	6187	
Money to own parents	0.000 (0.007)	0.016* (0.009)	0.538
Observations (*N*)	7125	6144	
Contraceptive use	−0.000 (0.006)	0.011** (0.005)	0.461
Observations (*N*)	7114	6177	
Baseline controls	No	Yes	
Heterogeneous trends	No	Yes	
Individual FE	Yes	Yes	
Month FE	Yes	Yes	

*Notes:* Standard errors are clustered at the municipality level and reported in parentheses (.). Baseline controls include a woman’s age, age squared, indigenous status, education level, number of children, the presence of her spouse during the interview, and municipality covariates like the homicide rate per 100,000 inhabitants and the introduction of domestic violence and divorce laws. Column (1) does not include any controls, and column (2) adds baseline controls and time trends interacted with municipal characteristics such as rural-urban status, transport network quality, community quality and the presence of *ejidos* (heterogeneous market trends). All specifications include individual and month fixed effects. ***p < 0.01 **p < 0.05 *p < 0.1

The benchmark specification in column (2) reveals that women living in gold endowed municipalities experienced an improvement in their decision-making power by about 0.01 (0.006/0.541) of a standard deviation when compared to the mean. Equivalently, this suggests that a two standard deviation increase in the price of gold results in a 0.02% increase in a woman’s decision-making power index. This effect appears to stem from an observed rise in women’s bargaining power over food expenditures, money given to the woman’s own parents and contraceptive use, where coefficient estimates for decision-making power over these particular goods in column (2) show an increase of 0.04, 0.06 and 0.04% respectively, with a two standard deviation increase in gold prices. Though the magnitude of these effects is considerably small, the findings nonetheless indicate that economic booms in male-dominated sectors like gold mining, do not necessarily worsen a woman’s decision-making power at home and could instead lead to an increase in their intra-household bargaining power over certain goods.

In order to check that these results are not spurious, I draw from a separate pooled cross-sectional data set, *ENDIREH*. As discussed in section III.a3, the *ENDIREH*, which was conducted in 2003, 2006 and 2011 also contains information on women’s decision-making power, though geographic data is only available on the state-level. Subsequently, I utilize this data set as a robustness check to examine if the gold mining boom indeed generated an increase in intra-household female bargaining power as reflected in the *MxFLS*.^21^ Since individuals in the *ENDIREH* however, were only interviewed over a period of one month between mid-October and mid-November for all three survey rounds, exploiting gold prices as a source of temporal variation is not feasible. Consequently, I define the post-treatment group as individuals interviewed in the 2006 and 2011 survey during which gold prices and production increased sharply, and those interviewed in 2003 as the pre-treatment group. Given that the *ENDIREH* surveyed individuals about their experiences with IPV and intra-household bargaining power 12 months (1 year) prior to the date of interview in the *ENDIREH*, responses in 2003 represent 2002 outcomes before gold prices started to rise in 2003. Table 10 of the appendix reports some descriptive statistics of the mean value of all covariates for both treatment and control groups in the *ENDIREH*. The summary statistics show that women in the control group are more likely to be rural and indigenous, have more children and have higher levels of education compared to the treated group. Given that the Mexican drug war only began in 2007, the large differences in the homicide rate between the treated and control group is expected as the control group was exposed to much less violence prior to 2007. The phase-in implementation of three domestic violence and divorce laws across states and time also explains the difference in mean values between the treated and control groups. For most of the covariates, the difference in means between both groups are statistically significant, indicating the importance of controlling for these covariates in the baseline regressions. Accordingly, I estimate the following difference-in-differences model:

2 $$Y_{i,s,t} = B_0 + B_1\left( {Gendowment_s \ast Post_t} \right) + B_2X_{i,t}^\prime + B_3Z_{s,t}^\prime + \emptyset _t + \pi _s + \varepsilon _{i,s,t}$$

Where *Y*_*i,s,t*_ *i*s woman *i*’s average decision-making power index in state *s* during survey year *t*, *Gendowment*_*s*_ is the log of the land area claimed by gold mining concessions between 1973 and 1983 in state *s*, *Post*_*t*_ is a binary variable equal to 1 for the survey years 2006 and 2011 during the peak of the gold price increase, and 0 otherwise. $X_{i,t}^\prime$ and $Z_{s,t}^\prime$ are vectors of individual- and state-level covariates such as a woman’s rural and indigenous status, age, age squared, length of relationship with her partner, number of children, education level, the homicide rate per 100,000 inhabitants, the timing of domestic violence and divorce law reforms across Mexican states and time trends interacted with state characteristics such as proximity to coast, protected areas, presence of *ejidos*, population density and physio geographic classification to account for heterogeneous market trends associated with prior state conditions. ∅_*t*_ and *π*_*s*_ is the survey year of interview and state fixed effects respectively, and *ε*_*i,s,t*_ is the error term. The results from estimating Eq. (2) are shown in Table 2.

**Table 2:** The effect of the mining boom on female decision-making outcomes (*ENDIREH* data)

	(1)	(2)	Mean (std. dev.)
DMP index (overall)	0.001 (0.001)	0.003** (0.001)	0.640
Observations (*N*)	191,768	186,506	
DMP index (HH expenditures)	0.004 (0.002)	0.011*** (0.002)	0.832
Observations (*N*)	190,246	185,107	
DMP index (who uses the contraception)	−0.004* (0.002)	−0.006*** (0.002)	0.622
Observations (*N*)	115,055	112,242	
DMP index (contraceptive use)	−0.001 (0.002)	−0.001 (0.002)	0.594
Observations (*N*)	116,173	113,328	
Baseline controls	No	Yes	
Heterogeneous trends	No	Yes	
State FE	Yes	Yes	
Survey year FE	Yes	Yes	

*Notes:* Standard errors are clustered at the state level and reported in parentheses (.). Baseline controls include a woman’s rural and indigenous status, age, age squared, length of relationship with her partner, number of children, education level, and state-level controls like the homicide rate per 100,000 inhabitants and the introduction of domestic violence and divorce laws. Column (1) does not include any controls, and column (2) adds baseline controls and time trends interacted with state characteristics like proximity to coast, protected areas, presence of *ejidos*, population density and physio geographic classification (heterogeneous market trends). All specifications include state and survey year fixed effects. ***p < 0.01 **p < 0.05 *p < 0.1

As can be seen, the same qualitative conclusion from the *MxFLS* data set can be drawn, where the estimated coefficient of a woman’s overall decision-making power index is positive and statistically significant. In particular, women living in gold endowed states experienced increases in their aggregate decision-making power by 0.0005 (0.003/0.640) of a standard deviation relative to the mean. Similar to the findings from the *MxFLS*, this result appears to be driven particularly by household expenditures where women’s decision-making power over this category of goods increased by about 0.013 (0.011/0.832) of a standard deviation. The findings using the *ENDIREH* data set in Table 2 also indicate that women in gold endowed states experienced lower bargaining power over the decision of *which partner should use contraception*, with estimated coefficients showing a decline in a woman’s decision-making authority over this matter by 0.001 (0.006/0.622) of a standard deviation when compared to the mean. For clarification, this questionnaire is distinct from the one in the *MxFLS* (Table 1) which asks women about the *general use of contraception*. The estimate for a woman’s bargaining power over this respective matter (general contraceptive use) using the *ENDIREH* in Table 2, reveals estimates that are statistically insignificant and zero-bound, different from the significant and positive estimate observed in Table 1 using the *MxFLS*. Overall, the effect of the gold mining boom on women’s bargaining power over general contraceptive use remains ambiguous, albeit there is some evidence pointing to a decline in their decision-making authority over *which partner* in the relationship should use contraception. The effect of the gold mining boom on the remaining decision-making categories which do not show a statistically significant relationship are presented in Table 11 of the appendix. Taken together, albeit the estimated coefficients from the analysis using both the *MxFLS* and *ENDIREH* are quantitatively small, they support the basic conclusion that the gold mining boom improved overall intra-household female decision-making power. Moreover, this effect was likely to be driven by the increase in bargaining power over household goods and services such as food expenditures.

### Possible channels

It is important to examine the effect of the gold mining boom on labor market outcomes and other determinants of welfare, to better understand the underlying mechanisms behind the increases in female decision-making power. Accordingly, Table 3 presents results from an analysis of the effect of the gold mining boom on male and female employment probabilities in the *MxFLS*. It also shows the impact of the gold mining boom on individuals’ perceptions of safety outside of the home given the reported association between drug-related violence and gold mines in Mexico (GIATOC, 2016). Across all specifications, the estimates reveal that men living in gold endowed municipalities experienced an increase in employment prospects during the mining boom. Female employment on the other hand, appears to have been unaffected. In particular, male employment probability estimates are statistically significant at the 10% level and positive in the baseline specification in (2), where the estimated coefficient shows an increase in male employment of 1.2 percentage points (or 1.5% when compared to the sample mean). Coefficients for female employment probabilities however, are insignificant and close to zero, indicating that while the gold mining boom had a positive impact on male employment, it did not have any effect on female labor force participation.

**Table 3:** The effect of mining on labor market outcomes and perceptions of safety (*MxFLS* data)

	(1)	(2)	Mean
*Men:*			
Employed	0.018*** (0.007)	0.012* (0.006)	0.803
Observations (*N*)	8420	6968	
Fear of assault	0.028* (0.016)	0.029* (0.016)	0.296
Observations (*N*)	8747	7042	
*Women:*			
Employed	0.003 (0.005)	0.001 (0.004)	0.190
Observations (*N*)	10,471	9131	
Fear of assault	0.017 (0.014)	0.014 (0.012)	0.370
Observations (*N*)	10,471	9131	
Baseline controls	No	Yes	
Heterogeneous trends	No	Yes	
Individual FE	Yes	Yes	
Month FE	Yes	Yes	

*Notes:* Standard errors are clustered at the municipality level and reported in parentheses (.). Baseline controls include a woman’s age, age squared, indigenous status, education level, number of children, the presence of her spouse during the interview, and municipality covariates like the homicide rate per 100,000 inhabitants and the introduction of domestic violence and divorce laws. An analogous set of covariates corresponding to men are included in the male sample. Column (1) does not include any controls, and column (2) adds baseline controls and time trends interacted with municipal characteristics such as rural-urban status, transport network quality, community quality and the presence of *ejidos* (heterogeneous market trends). All specifications include individual and month fixed effects. The differences in sample size between men and women is due to the fact that fewer men were interviewed in the *MxFLS* than women. ***p < 0.01 **p < 0.05 *p < 0.1

Altogether, these results suggest that the observed increase in female decision-making power was likely to be driven by increases in male employment during the gold mining boom. Unitary household bargaining models lend support to this finding as women’s intra-household decision-making power increased despite no observable changes in their employment probabilities. This indicates income pooling which is a central prediction of unitary models of the household, where the consumption of goods by the husband or wife depends solely on the total household income rather than on the sources of income. The same household income effect has also been documented in other developing countries like India (see Neff et al. 2012; Klasen and Pieters, 2015), where the elasticity of female labor supply increased in response to higher household wealth. Subsequently, since male employment in gold endowed municipalities increased, suggesting a rise in aggregate household income, women simply could have had more household wealth to allocate expenditures to household goods like food. In addition, the 2002 *MxFLS* pre-treatment survey wave shows that women had higher decision-making power over household goods and services compared to men (0.404 for women versus 0.176 for men), lending support to the idea that since women have conventionally had more control over household matters, the increase in women’s bargaining power over this particular set of goods and services could have simply been driven by an improvement in their husbands’ employment opportunities.

Table 3 also reveals some evidence of a decrease in men’s perception of safety in gold endowed municipalities during the boom, though there were no changes in women’s safety perceptions. The estimated coefficients for men’s fear of assault are significant and positive at the 10% level, and show that the gold mining boom increased men’s fear of getting assaulted outside of their home by 2.9 percentage points (or 10% when compared to the sample mean). This result is plausible, and could possibly be explained by the increase in drug-related violence in gold endowed municipalities as some Mexican drug cartels reportedly switched into gold production, and since mining is still predominantly a male economic sector (GIATOC, 2016). Men in gold endowed municipalities were therefore likely to be exposed to more dangerous surroundings as a result. Subsequently as expected, the baseline regressions show a strong and significant positive correlation between men’s fear of assault and the municipal homicide rate. A first-stage regression analysis of the relationship between the homicide rate and the interaction term of gold prices and endowment, also reveal statistically significant *t*-statistics (2.86), indicating a positive relationship between the gold mining boom and violence.^22^ Lastly, these findings also provide evidence of the male-breadwinner stereotype, where despite men being more afraid of getting assaulted or attacked during the gold mining boom, male employment probabilities still increased, with no observable changes in the female labor supply.

### Additional outcome: intimate partner violence (IPV)

Since the state-level *ENDIREH* data set also contains information on women’s experiences of intimate partner violence (IPV), I examine the effect of the gold mining boom on several types of gender-based violence as an additional outcome in the study. Following Bobonis et al.’s (2013) approach, IPV incidences are grouped into the four main categories: physical, sexual and emotional abuse, and the threat of violence, with a total of eight questions classified under physical violence, three questions under sexual violence, thirteen questions under emotional violence, and two questions under the threat of violence.^23^ For physical and sexual violence, I create dichotomous variables equal to one if a woman experienced a single incident in the past year. Considering that emotional violence is more likely to be subject to personal interpretation, I construct an emotional violence indicator equal to one if a woman answered “yes” to one incident, but stated that it happened multiple times, or if she answered “yes” to at least two emotional abuse questions. Lastly, the threat of violence indicator is equal to unity if a woman answered “yes” to at least one threat of violence question.

Accordingly, I estimate the same equation in (2), but replace the dependent variable with dichotomous indicators of the four different types of IPV. The results from the analysis are displayed in Table 4, and show that while women in gold endowed states had increased decision-making power at home, they were also more likely to suffer from all four forms of IPV. Specifically, the benchmark specification in column (2) shows that women living in gold endowed states experienced approximately 0.9, 0.1, 0.3 and 0.4 percentage points more emotional abuse, threats of violence, physical abuse and sexual abuse respectively.^24^ Compared to the mean values during the pre-treatment period before the gold mining boom, these figures represent an average increase in all four types of IPV by approximately 3.0% (emotional abuse), 3.4% (threats of violence), 3.8% (physical abuse) and 5.5% (sexual abuse). In a cross-country study on male-female job opportunities and IPV, Bhalotra et al. (2020) documented similar effect sizes of female unemployment on the incidence of physical violence. In their paper, a 1% increase in female unemployment was associated with a 2.75 and 2.87% increase in physical abuse respectively.

**Table 4:** The effect of the gold mining boom on intimate partner violence (IPV) (*ENDIREH* data)

	(1)	(2)	Mean
Emotional Abuse	0.002 (0.002)	0.009*** (0.002)	0.297
Observations (*N*)	168,787	163,902	
Threat of Violence	0.001** (0.001)	0.001** (0.001)	0.029
Observations (*N*)	191,940	186,666	
Physical Abuse	0.002** (0.001)	0.003** (0.001)	0.080
Observations (*N*)	192,011	186,730	
Sexual Abuse	0.003*** (0.001)	0.004*** (0.001)	0.073
Observations (*N*)	191,665	186,413	
Baseline controls	No	Yes	
Heterogeneous trends	No	Yes	
State FE	Yes	Yes	
Survey year FE	Yes	Yes	

*Notes:* Standard errors are clustered at the state level and reported in parentheses (.). Baseline controls include a woman’s rural and indigenous status, age, age squared, length of relationship with her partner, number of children, education level, and state-level controls like the homicide rate per 100,000 inhabitants and the introduction of domestic violence and divorce laws. Column (1) does not include any controls, and column (2) adds baseline controls and time trends interacted with state characteristics like proximity to coast, protected areas, presence of *ejidos*, population density and physio geographic classification (heterogeneous market trends). All specifications include state and survey year fixed effects. ***p < 0.01 **p < 0.05 *p < 0.1

The positive relationship between the gold mining boom and women’s risk of domestic violence could potentially be a result of male psychological stresses related to increased levels of fear. As shown in Table 3, the gold mining boom had a negative impact on men’s perception of safety, which was likely due to increased drug-related crime in mining areas. These findings can therefore be juxtaposed against several studies by Angelucci (2008) and Heise and Kotsadam (2015) for instance, that underscore psychological stress and trauma as drivers of domestic abuse. The rise in female intra-household decision-making power along with a concurrent increase in domestic violence is a striking finding, which could also possibly be explained by theories on male-backlash. The male-backlash effect, first proposed by Macmillan and Gartner (1999) suggests that when a wife experiences an increase in her tangible or intangible independence, men may be stimulated to perpetrate more violence as a means of “reinstating dominance and authority over his wife.” Therefore, in order to emotionally compensate for the increase in women’s decision-making power at home despite better employment prospects, men may retaliate through various forms of IPV.

Notwithstanding, given that women’s IPV outcomes are from a more spatially aggregated analysis (state-level) compared to decision-making outcomes conducted at the municipality level, one should remain cautious about attributing the rise in women’s IPV risk solely to these mechanisms related to the gold mining boom. Because more spatially disaggregated data on IPV in Mexico is not available, it is not feasible to check if the mechanisms that happen at a more localized level, like at the municipality level in the *MxFLS* for example, are comparable to those that occur at a more aggregated level. Lastly, in order to provide a more detailed breakdown of the effect of the gold mining boom on IPV, I create a dummy for each individual question on physical abuse, sexual abuse, emotional abuse and the threat of violence, and conduct an analysis analogous to Eq. (2). Results from the analysis are presented in Tables 13–16 of the appendix.

In a subsequent analysis, I examine the impact of the gold mining boom on women’s IPV outcomes across various socio-economic groups, as wider educational gaps between women and their spouses have been documented to increase IPV risk (Hidrobo et al., 2016; Heath, 2014). Additionally, because rural areas may have weaker policing, or have greater proportions of indigenous populations where the *machismo* culture is likely to be stronger, women residing in secluded places may also be subject to more IPV than those living in more urbanized areas. To test for the presence of such heterogeneous effects, I divide women from the *ENDIREH* into four sub-samples according to their education level (low or high) and their residential status (rural or urban).^25^ Accordingly, I examine the effect of the gold mining boom on each of the four sub-samples of women by repeating the estimation in Eq. (2), with the results presented in Table 5. The findings show that the indicator for physical violence is positive and significant for women living in rural areas and those with low education levels, where the probability of physical abuse is approximately 0.7 and 0.4 percentage points (8.4 and 4.8% in comparison to the mean) higher for rural women, and low educated women respectively. In addition, the results reveal that women from lower socio-economic classes were also more likely to experience emotional abuse with estimated coefficients significant and positive across the two different groups of women (1 and 0.8 percentage points for rural and low educated women respectively). Overall, less educated women appear to suffer more from sexual abuse, facing an increase in the probability of this type of abuse by about 0.7 percentage points.

**Table 5:** The effect of mining on the incidence of intimate partner violence (IPV) among women from lower- and upper- socio-economic classes (*ENDIREH* data)

	Rural	Urban	Low- educated	High- educated
Emotional	0.010***	0.007***	0.008*	0.012***
Abuse	(0.003)	(0.002)	(0.004)	(0.004)
Mean	0.265	0.306	0.296	0.298
Observations (*N*)	33,527	130,375	105,714	58,607
Threat of	−0.002	0.002***	0.002	0.003**
Violence	(0.001)	(0.001)	(0.001)	(0.001)
Mean	0.030	0.029	0.034	0.024
Observations (*N*)	37,430	149,236	120,203	66,918
Physical	0.007**	0.001	0.004**	0.001
Abuse	(0.003)	(0.001)	(0.002)	(0.001)
Mean	0.083	0.078	0.084	0.074
Observations (*N*)	37,435	149,295	120,234	66,951
Sexual	0.002	0.004***	0.007***	0.002
Abuse	(0.002)	(0.001)	(0.002)	(0.002)
Mean	0.081	0.071	0.087	0.057
Observations (*N*)	37,372	149,041	120,027	66,837
Baseline controls	Yes	Yes	Yes	Yes
Heterogeneous trends	Yes	Yes	Yes	Yes
State FE	Yes	Yes	Yes	Yes
Survey year FE	Yes	Yes	Yes	Yes

*Notes:* Standard errors are clustered at the state level and reported in parentheses (.). Baseline controls include a woman’s rural and indigenous status, age, age squared, length of relationship with her partner, number of children, education level, and state-level controls like the homicide rate per 100,000 inhabitants and the introduction of domestic violence and divorce laws. Heterogeneous trends include time trends interacted with state characteristics like proximity to coast, protected areas, presence of *ejidos*, population density and physio geographic classification. All regressions include baseline controls, heterogeneous market trends, state fixed effects and survey year fixed effects. Sample sizes differ due to the difference in the number of individuals belonging to each sub-sample. ***p < 0.01 **p < 0.05 *p < 0.1

Next, I estimate the impact of the mining boom on women from upper socio-economic classes: those who live in urban areas and are relatively more educated. The results reveal that the positive gold mining shock did not have any impact on the probability of physical abuse for women from upper socio-economic classes. In particular, the coefficients for the physical abuse indicator for women living in urban areas and who are highly educated are statistically insignificant and close to zero. These women however, appear to suffer more from emotional abuse and threats of violence than their poorer counterparts. The findings show that urban and high educated women experienced a 0.7 and 1.2 percentage point (2.3 and 4% respectively when compared to the mean) increase in their likelihood of emotional abuse, and urban and high educated women were subject to 0.2 and 0.3 percentage points (6.9 and 12.5% respectively) more threats of violence from their partners respectively. The results also indicate that urban women are more likely to suffer from sexual abuse than high educated women, with a 0.4 percentage point (5.6%) increase in their probability of experiencing this type of IPV.

To explain why poorer women may be more susceptible to harder forms of IPV like physical abuse, I draw from a pioneering study by Gelles (1976) which showed that women who had fewer financial resources were less likely to leave an abusive relationship. Given that poorer women may be more hesitant to report domestic violence or file for divorce due to their lower income earning potential, and hence greater reliance on their husbands’ incomes, men with partners from lower socio-economic classes may be less wary about exhibiting harder types of IPV like physical or sexual abuse. On the other hand, women from upper socio-economic classes may be more likely to report domestic violence or file for divorce in cases of wife abuse, given their greater economic independence and earning potential. Partners of women from higher socio-economic classes may thus commit softer forms of IPV that are less tangible in nature and harder to prosecute.

#### Robustness: sensitivity analyses and alternative measures of DMP

To further probe the robustness of the main results, I use an alternative empirical model to examine the effect of the gold mining boom on female decision-making power. Specifically, I estimate a multinomial logit fixed effects model where the decision-making power indicator is a categorical dependent variable ranging from 1 to 3, with the same covariates and fixed effects as in Eq. (1). For instance, if a woman responds that she was the sole decision-maker of that particular good category, it is assigned a value of 3 (woman has full power in decision-making). If the decision was jointly made with her husband, the category is assigned a value of 2, and if the decision was made solely by her husband, the category is assigned a value of 1 (indicating that the woman had no bargaining power over that good at all). The results from the analysis are presented in Table 17 of the appendix, which show qualitatively similar results as Table 1. Using value 3 (woman has full decision-making power) as the base comparison category, the findings indicate that with a unit increase in the decision-making power over the money given to a woman’s spouse’s parents ceteris paribus, the logarithm of the probability that the husband makes the full decision (decision is jointly made) in this category relative to the wife making the full decision in this category decreases by 0.246 (0.248). Equivalently, decision-making over food expenditures is only statistically significant for the joint decision-making category and shows that the odds of the decision being made jointly in this category versus the decision being made fully by the wife decreases by 0.088. Coefficient estimates are not significant for other categories of goods and services. In essence, the results point to the same baseline conclusion that women living in gold endowed municipalities were more likely to partake in intra-household decision-making processes compared to those that did not.

As an additional robustness check, I conduct a series of sensitivity analyses on the main female decision-making power estimates presented in Table 1. First, I omit municipalities in three Northern states: Oaxaca, Chihuahua and Durango where gold mining activity is the most prominent (see Fig. 2) to ensure that the main results are not driven by mining intensive states in this region of Mexico’s geographical landscape. Altogether, the results presented in Table 6 show that the baseline estimate in column (2) of Table 1 increased by 0.002 to 0.007 compared to the main estimate of 0.005, with coefficients remaining significant at the 5% level. Next, I exclude municipalities with high levels of violence, specifically those in the 75^th^ percentile according to the homicide rate. I do so to check if more violent municipalities downward biased the main female decision-making power estimates given the positive correlation between gold mining and drug-related violence, and subsequently, the documented negative effect of violence on women’s intra-household bargaining power (see Tsaneva et al. 2018). The results from this analysis provide some indication that the main coefficient of a woman’s decision-making power in Table 1 is underestimated, as excluding violent municipalities increases the estimated coefficient by 0.001 when compared to the baseline estimate of 0.005. This therefore suggests that in the absence of the confounding drug war, women residing in gold endowed municipalities would have had higher intra-household decision-making power than reported in Table 1.

**Table 6:** Sensitivity analysis of effect of the gold mining boom on female decision-making power (overall) (*MxFLS* data)

	(1)	(2)	Mean
Exclude northern municipalities	0.001 (0.004)	0.007** (0.003)	0.539
Observations (*N*)	9225	8020	
Exclude violent municipalities	0.003* (0.002)	0.006** (0.002)	0.540
Observations (*N*)	7580	6763	
HR*Gendowment*Price	0.000 (0.001)	0.004* (0.003)	0.541
Observations (*N*)	10,462	9,123	
World export value of gold	0.013* (0.007)	0.016* (0.008)	0.548
Observations (*N*)	7786	4664	
Baseline controls	No	Yes	
Heterogeneous trends	No	Yes	
Individual FE	Yes	Yes	
Month FE	Yes	Yes	

*Notes:* Standard errors are clustered at the municipality level and reported in parentheses (.). Baseline controls include a woman’s age, age squared, indigenous status, education level, number of children, the presence of her spouse during the interview, and municipality covariates like the homicide rate per 100,000 inhabitants and the introduction of domestic violence and divorce laws. Column (1) does not include any controls, and column (2) add baseline controls and time trends interacted with municipal characteristics such as rural-urban status, transport network quality, community quality and the presence of *ejidos* (heterogeneous market trends). All specifications include individual and month fixed effects. ***p < 0.01 **p < 0.05 *p < 0.1

In a following robustness check, I include the interaction term of homicide rates, gold endowment and world gold prices. Albeit the movement of drug cartels into gold mining reportedly only intensified from 2012 onwards according to GIATOC (2016), one cannot rule out that the spike in gold mining opportunities during the sample period prior to 2012, was also accompanied by an increase in drug-related crime. Since excluding violent municipalities increases the main decision-making power estimate, and given the evidence presented in Table 3 reflecting decreased perceptions of safety among men in the *MxFLS*, it is important to account for the possible conflicting impact that drug-related violence could have had on female intra-household bargaining outcomes during the gold mining boom. Subsequently, Table 6 shows that the inclusion of the interaction term of drug-related crime proxied by homicide rates with gold endowment and price, decreases the statistical significance of the main estimate to the 10% level. The estimated coefficient of a woman’s overall mean decision-making power index also decreases, though minimally by 0.001 compared to the main coefficient of 0.005. Taken together, this indicates that the relationship between drug-related crime and the gold mining boom in Mexico was not likely to have affected female decision-making power outcomes significantly, at least during the sample period of the study. In addition, a number of papers namely by Brown et al. (2017), Ajzenman et al. (2015) and Velásquez (2010) have reaffirmed that differences in the economic impact of the global financial crisis were uncorrelated with differences in homicide growth rates across municipalities in Mexico.

As a final robustness test, I replace global gold prices with the world value of gold exports less Mexico, with data provided by the *International Trade Centre (ITC)*. Because the value of women’s assets increases along with the rising global gold prices if they possess gold jewelry for instance, this would improve their outside option, which could subsequently increase their intra-household decision-making power as predicted by non-unitary household bargaining models. The *ITC* however, only supplies trade data containing information on each country’s export value of gold from 2004 to present. As such, it is not feasible to use all three waves of the *MxFLS* for this analysis as no available data exists for the first wave conducted in 2002. Accordingly, I utilize two waves of the *MxFLS* conducted in 2005–2006 and 2009–2010, and match individuals’ month and year of interview to the world value of gold exports. Results from estimating an analogous regression to Eq. (1), replacing world gold prices with the world value of gold exports less Mexico is shown in Table 6. The estimates support the basic conclusion that women residing in gold endowed municipalities experienced increases in their decision-making power. Coefficients are nonetheless evidently larger, reflecting an increase of 0.011, up from the main estimate of 0.005, which could be due to the different sample and time frame analysed.

#### Robustness: parallel trends

**Fig. 3:**
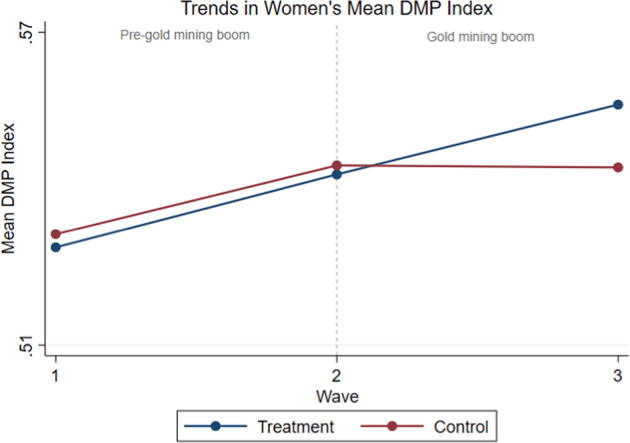
Trends in women’s mean decision-making power (DMP) index in the *MxFLS* across three survey waves (2002, 2005–2006, 2009–2010). *Notes*: This figure plots pre- (before the gold mining boom) and post- (during the gold mining boom) trends of women’s average decision-making power indices across all three *MxFLS* waves. The blue (red) line denotes the treatment (control) group

**Fig. 4:**
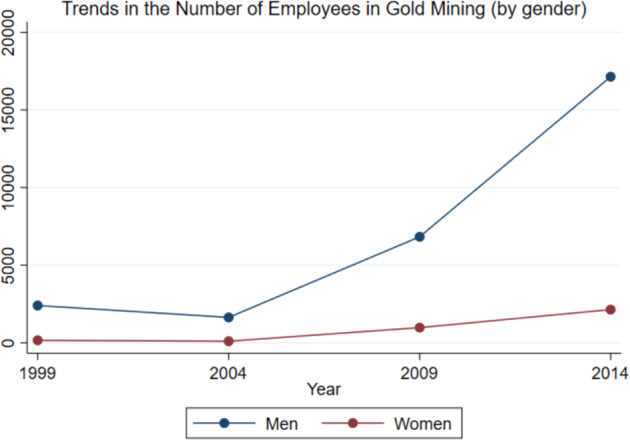
Trends in the number of employees in gold mining, by gender. *Notes*: This diagram shows the trends in the number of male (blue) and female (red) employees hired in gold mining between 1999 and 2014. Data is from the National Institute for Statistics and Geography (INEGI), and is only available for the years 1999, 2004, 2009 and 2014 as the economic census is only conducted once every 5 years

**Fig. 5:**
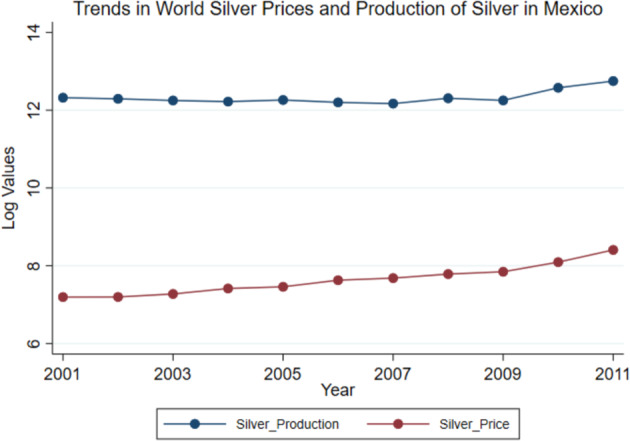
Trends in world silver prices and Mexican silver production. *Notes*: This diagram shows the relationship between world silver prices and the production of silver in Mexico between 2001 and 2011. Each point is the logged value of prices or production. The blue (red) line denotes production (price). Data on Mexico’s silver production come from the National Institute for Statistics and Geography (INEGI), and data on world silver prices is from the London Metal Exchange’s (LME) monthly historical price series

**Fig. 7:**
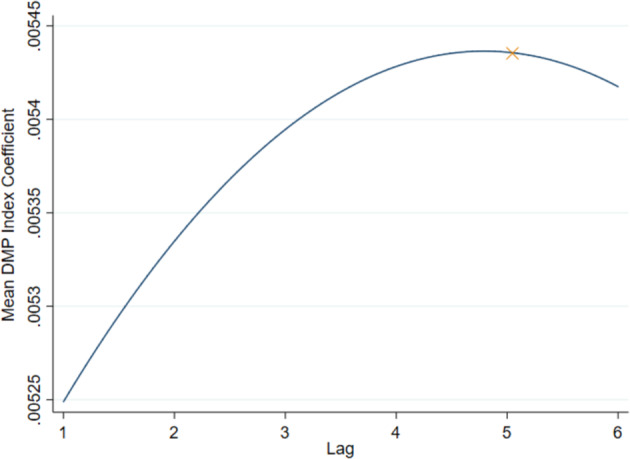
Coefficient plot for a woman’s relative decision-making power (DMP). *Notes*: This figure plots the coefficients of the regression of a woman’s decision-making power index on the lag of gold prices (*t* = 1, 2,..,6). The orange ‘x’ denotes the highest coefficient value at *t-5*, and shows a quadratic relationship between lagged price proxy values and a woman’s decision-making power estimate

The difference-in-differences estimation method hinges on the assumption that in the absence of the gold mining boom, the decision-making outcomes for women in the treatment group (those living in gold endowed municipalities), and women in the control group (women who do not live in gold mining municipalities) follow parallel trends. Accordingly, I plot the average decision-making power index of women in both groups across all three *MxFLS* survey waves in Fig. 3. The graph provides some support for the common trends assumption required for a difference-in-differences analysis as it indicates that trends in women’s average decision-making power index for both the treatment and control group were on similar paths during the pre-gold mining boom period (wave 1 to 2). Moreover, Fig. 3 shows a divergence in women’s decision-making power trends between the treatment and control group during the gold mining boom (wave 2 to 3), with women in the treated (control) group experiencing higher (lower) average decision-making power during this time period. This observation is consistent with the main estimates presented in Table 1, showing that women who resided in gold endowed municipalities experienced higher decision-making power at home.

## Discussion and concluding remarks

How accurate are theories that postulate a decrease in female decision-making power during booms in male-dominated sectors like gold mining? One existing study by Tolonen (2018) which focuses on Sub-Saharan Africa, did not discover any effects of gold mining on women’s intra-household decision-making outcomes. Using Mexico’s gold mining boom which was stimulated by the 2007–2008 global financial crisis as an exogenous event, I show that women residing in gold endowed municipalities experienced small but statistically significant increases in their average decision-making power over a set of goods and services, against the theoretical predictions by non-unitary household bargaining models. The results are robust to the inclusion of heterogeneous market trends, an alternative decision-making power index, various sensitivity analyses, and are consistent with findings from a separate state-level survey (*ENDIREH*) containing similar questionnaires on spousal decision-making.

The rise in female decision-making authority is likely to have been driven by the increase in male employment during the gold mining boom, but not female employment as no changes in female employment probabilities were observed. These findings are consistent with unitary household bargaining models, which predict that regardless of how much each individual contributes to household income, the outcomes of household decisions only depend on the aggregate household wealth. The results also provide support for the income pooling assumption of the unitary household bargaining framework, as women still experienced increases in their decision-making power, particularly over household goods like food expenditures for instance, despite not working more. This indicates that financial gains from the increase in male employment was likely to have been allocated to household expenditures, which women have traditionally been more responsible for.

Using an alternative state-level data set (*ENDIREH*) containing information on women’s IPV experiences, I additionally find that although women living in gold endowed states experienced higher intra-household bargaining power, they were also more likely to suffer from four different types (physical, sexual, and emotional abuse, and threats of violence) of IPV. Particularly, poorer women were more susceptible to hard forms of IPV like physical abuse, and more well-off women suffered from softer forms of IPV like threats of violence and emotional abuse. These findings could possibly be attributed to various factors such as men’s heightened fear of assault and psychological stress possibly due to drug-related crime in gold mining areas, differences in poor and rich women’s responses and acceptance towards domestic violence and the male-backlash effect. A limitation of the data on IPV however, is that location information which is only available at the state-level is not as disaggregated as that of the *MxFLS* panel which contains municipality data. Therefore, since the regression for IPV outcomes is not as tightly controlled as those for decision-making power using the *MxFLS*, caution is required when attributing any effects on IPV solely to the gold mining boom. Notwithstanding, since the *ENDIREH* is the only Mexican survey that has extensively surveyed women across the country about their IPV experiences, the results arguably still shed some light, more broadly, on the effects of changing economic conditions on women’s probability of IPV in the context of a Latin American country like Mexico.

From a policy perspective, this study underscores the need for more work in altering social and gender norms to accommodate increases in intra-household female decision-making power. In particular, it is important for gender mainstreaming efforts to specifically target the balancing of male psychological and emotional shifts along with increases in both tangible and intangible aspects of female empowerment. For instance, efforts should focus on providing more counseling services for men facing stressful periods, and in mitigating the male-backlash effect when men’s authority come under threat. Doing so could help to ensure that an increase in a woman’s bargaining power would not be accompanied by more IPV as demonstrated in this study. More generally, this paper highlights the importance of investing more in women shelters that guarantee protection, rather than substitute them with policies that may backfire, such as giving cash to victims of domestic violence (El Congreso del Estado de Michoacán 2019). Additionally, cash transfers specifically in Mexico have been found to be associated with higher rates of verbal and emotional abuse such as threats of violence (Bobonis et al. 2013). In line with the unitary household framework, the evidence from this study thus suggests that irrespective of whether the husband or wife receives the cash transfer, domestic violence will increase as aggregate household income increases. In this context, cash transfers may therefore not be the optimal solution for protecting women from violence, as while it may improve a woman’s household decision-making power, it could also potentially increase her risk of IPV. Instead, the adequate provision of female shelters in places where women are more vulnerable to domestic violence like in mining areas for instance, could be more useful in mitigating IPV.

What is the external validity of the results presented in this study? Due to socio-cultural differences, the findings from this paper may be more generalizable to mineral-rich countries in Latin America such as Colombia, Peru, Argentina, Brazil and Ecuador, rather than Africa, where little evidence of the impact of mining on female bargaining power and IPV was found (Kotsadam et al. 2016; Tolonen 2018). It may also be interesting for future research to examine the heterogeneous effects of different mining minerals on the various female welfare outcomes explored in this study. For example, oil extraction could have different effects on women’s intra-household well-being if the underlying mechanisms are distinct from the ones presented in this paper.

## Figures and Tables

**Fig. 6:**
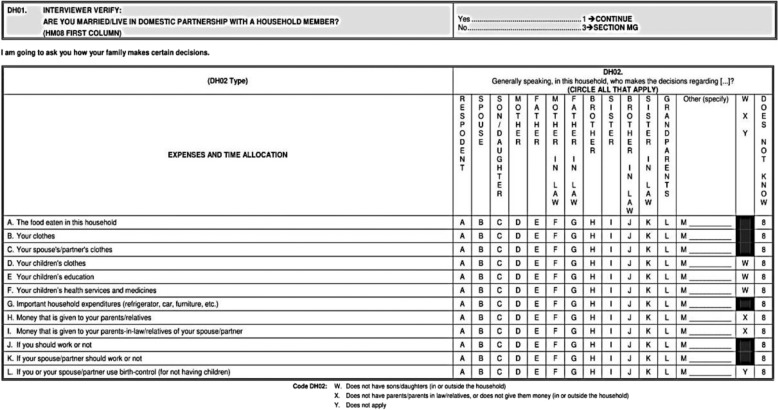
*MxFLS* questionnaire on decision-making. *Notes*: This diagram shows a sample of the decision-making questionnaire of the *MxFLS* survey. In total, twelve decision-making questions (‘A’ to ‘L’) are administered to both a woman and her partner. The decision-making questionnaire is the same across the two survey waves used in this study

## Appendix A: Figures

Figures 4–7

## Appendix B: Tables

Tables 7–17

**Table 7:** Summary statistics of dependent variables

	Mean	SD	Min	Max	*N*
*Dependent Variable–Woman’s average decision-making power (DMP) over all goods:*					
Mean DMP index (*MxFLS* data)	0.5	0.13	0	1	10,470
Mean DMP index (*ENDIREH* data)	0.7	0.21	0	1	191,768
*Dependent Variables–Woman’s decision-making power over (individual decisions) (MxFLS data):*					
Contraceptive use	0.5	0.3	0	1	7992
Spouse’s work choice	0.2	0.3	0	1	10,397
Own work choice	0.6	0.4	0	1	10,369
Money given to spouse’s family	0.3	0.3	0	1	7833
Money given to own family	0.5	0.4	0	1	7927
Large expenditures	0.4	0.3	0	1	10,295
Child’s health	0.5	0.3	0	1	8668
Child’s education	0.5	0.3	0	1	8469
Child’s clothes	0.6	0.4	0	1	7192
Spouse’s clothes	0.3	0.4	0	1	10,409
Own clothes	0.8	0.3	0	1	10,430
Food eaten in the house	0.8	0.3	0	1	10,388
*Other Dependent Variables (MxFLS data):*					
Male employment	0.8	0.4	0	1	8964
Female employment	0.2	0.4	0	1	10,478
Male fear of assault	0.3	0.5	0	1	9069
Female fear of assault	0.4	0.5	0	1	10,478
*IPV outcomes (ENDIREH data):*					
Emotional Abuse	0.3	0.5	0	1	168,787
Physical Abuse	0.1	0.3	0	1	192,011
Sexual Abuse	0.1	0.2	0	1	191,665
Threat of Violence	0.0	0.2	0	1	191,940

*Notes:* Summary statistics of dependent variables. The number of observations (*N*) for decision-making power questions vary, since not all women in the sample have children, and some decisions are neither made by the husband or the wife

**Table 8:** Summary statistics of independent variables

	Mean	SD	Min	Max	*N*
*Independent Variables (MxFLS data):*					
Indigenous	0.1	0.3	0	1	10,478
Presence of spouse during interview	0.1	0.3	0	1	10,455
Age	43.6	12.9	15	101	7990
Age squared	2074	1253	225	10,201	7990
Years of education	9.6	4.3	0	22	10,466
Number of children	1.6	1.5	0	9	10,478
Homicide rate	0.5	0.6	0	3.4	10,478
Assist Law	0.7	0.4	0	1	10,478
Divorce law	0.7	0.5	0	1	10,478
Penal Code	0.9	0.3	0	1	10,478
*Independent Variables (ENDIREH data):*					
Rural status	0.2	0.4	0	1	192,055
Indigenous	0.1	0.2	0	1	191,711
Age	40.9	13.8	15	104	191,849
Age squared	1860.1	1253.3	225	108,816	191,849
Length of relationship	21.3	14.0	1	83	189,142
Number of children	3.3	2.3	0	25	190,410
Homicide rate	15.9	19.4	2.0	130.6	192,055
Assist Law	0.8	0.4	0	1	192,061
Divorce law	0.8	0.4	0	1	192,061
Penal Code	0.9	0.3	0	1	192,061

*Notes:* Summary statistics of independent variables

**Table 9:** The effect of the gold mining boom on female decision-making power (other goods and services) (*MxFLS* data)

	(1)	(2)	Mean (std. dev.)
*Normalized DMP (individual goods):*			
Own clothes	−0.002 (0.005)	−0.002 (0.007)	0.829
Observations (*N*)	10,419	9087	
Spouse’s clothes	−0.007 (0.008)	−0.007 (0.010)	0.538
Observations (*N*)	7125	6144	
Child’s clothes	0.000 (0.008)	0.003 (0.012)	0.461
Observations (*N*)	5880	5092	
Child’s education	0.009 (0.007)	0.008 (0.006)	0.516
Observations (*N*)	7779	6774	
Child’s health	0.003 (0.010)	0.007 (0.009)	0.534
Observations (*N*)	8049	7014	
Spouse’s work choice	−0.006 (0.009)	−0.001 (0.010)	0.208
Observations (*N*)	10,389	9053	
Own work choice	0.004 (0.006)	0.008 (0.006)	0.594
Observations (*N*)	10,358	9026	
Money given to spouse’s parents	−0.003 (0.010)	0.008 (0.008)	0.292
Observations (*N*)	7000	6029	
Household expenditures	0.004 (0.006)	0.011 (0.007)	0.386
Observations (*N*)	10,266	8953	
Baseline controls	No	Yes	
Heterogeneous trends	No	Yes	
Individual FE	Yes	Yes	
Month FE	Yes	Yes	

*Notes:* Standard errors are clustered at the municipality level and reported in parentheses (.). Baseline controls include a woman’s age, age squared, indigenous status, education level, number of children, the presence of her spouse during the interview, and municipality covariates like the homicide rate per 100,000 inhabitants and the introduction of domestic violence and divorce laws. Column (1) does not include any controls, and column (2) adds baseline controls and time trends interacted with municipal characteristics such as rural-urban status, transport network quality, community quality and the presence of *ejidos* (heterogeneous market trends). All specifications include individual and month fixed effects. ****p* < 0.01 ***p* < 0.05 **p* < 0.1

**Table 10:** Descriptive statistics: *ENDIREH* survey

	*Post*_*t*_ = 1 (T)	*Post*_*t*_ = 0 (C)
*Individual Covariates:*		
Rural	0.196	0.229
Indigenous	0.059	0.092
Length of relationship	21.439	20.708
Age	41.072	39.875
Age squared	1877.746	1769.333
Number of kids	3.181	3.624
Education level	3.579	4.145
*State Covariates:*		
Homicide rate	17.366	8.728
Assist law	0.867	0.589
Divorce law	0.861	0.518
Penal Code	0.894	0.822
*Observations*	160,750	31,305

*Notes:* ‘T’ represents the treated group belonging to the 2006 and 2011 *ENDIREH* survey and ‘C’ represents the control group surveyed in 2003. The table reports mean values for each covariate and group

**Table 11:** The effect of the gold mining boom on female decision-making power (other goods and services) (*ENDIREH* data)

	(1)	(2)	Mean (std. dev.)
*Normalized DMP (individual goods):*			
Own work choice	0.000 (0.002)	0.004 (0.002)	0.829
Observations (*N*)	171,794	167,236	
Expenditures on own goods	0.003 (0.002)	0.000 (0.002)	0.538
Observations (*N*)	187,334	182,193	
Children’s goods	0.001 (0.001)	0.001 (0.001)	0.461
Observations (*N*)	162,143	158,935	
Baseline controls	No	Yes	
Heterogeneous trends	No	Yes	
State FE	Yes	Yes	
Survey year FE	Yes	Yes	

*Notes:* Standard errors are clustered at the municipality level and reported in parentheses (.). Baseline controls include a woman’s age, age squared, indigenous status, education level, number of children, the presence of her spouse during the interview, and state controls like the homicide rate per 100,000 inhabitants. Column (1) does not include any controls, and column (2) adds baseline controls and heterogeneous market trends. All specifications include state and survey year fixed effects. *** *p* < 0.01 ** *p* < 0.05 * *p* < 0

**Table 12:** The effect of the gold mining boom on intimate partner violence (IPV) with wild-cluster bootstrapped standard errors (*ENDIREH* data)

	(1)	(2)	Mean
Emotional Abuse	0.002 [0.004]	0.007** [0.008]	0.297
Observations (*N*)	168,787	163,902	
Threat of Violence	0.001** [0.001]	0.001* [0.001]	0.029
Observations (*N*)	191,940	186,666	
Physical Abuse	0.002** [0.001]	0.003** [0.003]	0.080
Observations (*N*)	192,011	186,730	
Sexual Abuse	0.003*** [0.002]	0.004*** [0.003]	0.073
Observations (*N*)	191,665	186,413	
Baseline controls	No	Yes	
Heterogeneous trends	No	Yes	
State FE	Yes	Yes	
Survey year FE	Yes	Yes	

*Notes:* Standard errors are clustered at the state level and reported in parentheses (.). Baseline controls include a woman’s age, age squared, indigenous status, education level, number of children, the presence of her spouse during the interview, and state-level controls like the homicide rate per 100,000 inhabitants. Column (1) does not include any controls, and column (2) adds baseline controls and heterogeneous market trends. All specifications include state and survey year fixed effects. *** *p* < 0.01 ** *p* < 0.05 * *p* < 0.1

**Table 13:** The effect of mining on the incidence of physical intimate partner violence (IPV) (*ENDIREH* data)

	(1)	(2)	Mean
Pushed you/Pulled your hair	0.001** (0.001)	0.002 (0.001)	0.061
Observations (*N*)	191,999	186,720	
Tied you up	−0.000* (0.001)	−0.000 (0.000)	0.002
Observations (*N*)	191,961	186,684	
Kicked you	0.001** (0.001)	0.001 (0.001)	0.019
Observations (*N*)	191,976	186,698	
Hit you with his hands	0.003*** (0.001)	0.002** (0.001)	0.047
Observations (*N*)	191,979	186,702	
Baseline controls	No	Yes	
Heterogeneous trends	No	Yes	
State FE	Yes	Yes	
Survey year FE	Yes	Yes	

*Notes:* Standard errors are clustered at the state level and reported in parentheses (.). Baseline controls include a woman’s age, age squared, indigenous status, education level, number of children, the presence of her spouse during the interview, and state-level controls like the homicide rate per 100,000 inhabitants. Column (1) does not include any controls, and column (2) adds baseline controls and heterogeneous market trends. All specifications include state and survey year fixed effects. *** *p* < 0.01 ** *p* < 0.05 * *p* < 0.1

**Table 14:** The effect of mining on the incidence of sexual intimate partner violence (IPV) (*ENDIREH* data)

	(1)	(2)	Mean
Forced sexual relations	0.001** (0.001)	0.001* (0.000)	0.022
Observations (*N*)	191,607	186,359	
Demand sex	0.003*** (0.001)	0.004*** (0.000)	0.069
Observations (*N*)	191,648	186,397	
Used force to have sex	0.002*** (0.001)	0.002*** (0.000)	0.023
Observations (*N*)	191,530	186,287	
Baseline controls	No	Yes	
Heterogeneous trends	No	Yes	
State FE	Yes	Yes	
Survey year FE	Yes	Yes	

*Notes:* Standard errors are clustered at the state level and reported in parentheses (.). Baseline controls include a woman’s age, age squared, indigenous status, education level, number of children, the presence of her spouse during the interview, and state-level controls like the homicide rate per 100,000 inhabitants. Column (1) does not include any controls, and column (2) adds baseline controls and heterogeneous market trends. All specifications include state and survey year fixed effects. *** *p* < 0.01 ** *p* < 0.05 * *p* < 0.1

**Table 15:** The effect of mining on the incidence of the threat of violence (IPV) (*ENDIREH* data)

	(1)	(2)	Mean
Threatened with weapon	0.001** (0.000)	0.001* (0.000)	0.014
Observations (*N*)	191,958	186,684	
Threatened to kill you	0.001** (0.001)	0.001 (0.001)	0.025
Observations (*N*)	191,943	186,668	
Baseline controls	No	Yes	
Heterogeneous trends	No	Yes	
State FE	Yes	Yes	
Survey year FE	Yes	Yes	

*Notes:* Standard errors are clustered at the state level and reported in parentheses (.). Baseline controls include a woman’s age, age squared, indigenous status, education level, number of children, the presence of her spouse during the interview, and state-level controls like the homicide rate per 100,000 inhabitants. Column (1) does not include any controls, and column (2) adds baseline controls and heterogeneous market trends. All specifications include state and survey year fixed effects. *** *p* < 0.01 ** *p* < 0.05 * *p* < 0.1

**Table 16:** The effect of mining on the incidence of the emotional intimate partner violence (IPV) (*ENDIREH* data)

	(1)	(2)	Mean
Destroyed things in house	0.002** (0.001)	0.002** (0.001)	0.046
Observations (*N*)	191,965	186,692	
Threatened to leave	0.002** (0.001)	0.002** (0.001)	0.087
Observations (*N*)	191,973	186,700	
Not allowed to leave home	0.000 (0.000)	0.001* (0.000)	0.036
Observations (*N*)	191,967	186,964	
Accused you of cheating	0.001 (0.001)	0.003** (0.001)	0.069
Observations (*N*)	191,940	186,667	
Made you feel fearful	0.003*** (0.001)	0.002** (0.001)	0.082
Observations (*N*)	191,954	186,681	
Ignored you	0.003** (0.001)	0.003 (0.002)	0.096
Observations (*N*)	191,963	186,691	
Baseline controls	No	Yes	
Heterogeneous trends	No	Yes	
State FE	Yes	Yes	
Survey year FE	Yes	Yes	

*Notes:* Standard errors are clustered at the state level and reported in parentheses (.). Baseline controls include a woman’s age, age squared, indigenous status, education level, number of children, the presence of her spouse during the interview, and state-level controls like the homicide rate per 100,000 inhabitants. Column (1) does not include any controls, and column (2) adds baseline controls and heterogeneous market trends. All specifications include state and survey year fixed effects. *** *p* < 0.01 ** *p* < 0.05 * *p* < 0.1

**Table 17:** The effect of mining on female decision-making power (multinomial logit), with full decision-making power as the base category (*MxFLS* data)

	Husband makes full decision		Joint decision-making	
	(1)	(2)	(3)	(4)
*Decision-making power over:*				
Food Expenditures	−0.089 (0.076)	−0.084 (0.099)	−0.063* (0.034)	−0.088** (0.041)
Observations (*N*)	5504	4786	5504	4786
Money to spouse’s parents	−0.232** (0.102)	−0.246* (0.130)	−0.206** (0.102)	−0.248* (0.129)
Observations (*N*)	3784	3276	3784	3276
Baseline controls	No	Yes	No	Yes
Heterogeneous trends	No	Yes	No	Yes
Individual FE	Yes	Yes	Yes	Yes
Month FE	Yes	Yes	Yes	Yes

*Notes:* Standard errors are clustered at the municipality level and reported in parentheses (.). Baseline controls include a woman’s age, age squared, indigenous status, education level, number of children, the presence of her spouse during the interview, and municipality covariates like the homicide rate per 100,000 inhabitants and the introduction of domestic violence and divorce laws. Columns (1) and (3) do not include any controls, and columns (2) and (4) add baseline controls and time trends interacted with municipal characteristics such as rural-urban status, transport network quality, community quality and the presence of *ejidos* (heterogeneous market trends). All specifications include individual and month fixed effects. ****p* < 0.01 ***p* < 0.05 **p* < 0.1

## Footnotes

1 S and P Global (2019) estimates that the Latin American region receives approximately 28–30% of the global budget in mining explorations each year, with six mining powerhouses, Peru, Brazil, Chile, Argentina, Ecuador and Mexico accounting for the lion’s share (90%) of the region’s budget.

2 INEGI Data on the share of men and women in mining is only available for the years 1999, 2004, 2009 and 2014 since the economic census is only conducted once every five years.

3 For instance, in Latin America, the *machismo* culture is particularly strong and had origins during colonial times, where women were perceived as weak, naive, and had to be protected by men as they could not fend for themselves (Bridges 1980). *Machoism* is therefore often associated with an increased risk of intimate partner violence (IPV) perpetration, and implies that men of Latino origin engage in harmful behaviors such as drunkenness, violence and infidelity (Gutmann and Viveros 2005; Mancera et al. 2017).

4 Given Mexico’s economic ties and dependence on the U.S. as an export market, all economic sectors including mining took the hit during the 2007–2008 global financial crisis. This explains the slight fall in gold production between 2008 and 2009. Mining was however the first industrial sector in Mexico to recover from the crisis as the economy started to recover in 2009, which is depicted by the sharp increase in gold production and prices between 2009 and 2011 (Engineering and Mining Journal, 2011). Moreover, the panel *MxFLS* data used in this study was conducted in 2002, 2005–2006 and 2009–2010, which does not overlap with the dip in gold production between 2008–2009.

5 Data for the gold and silver price ratio was from the London Bullion Market Association (LBMA) and ICE Benchmark Administration (IBA).

6 Estimation output for the regression is not listed; but available upon request.

7 Open-pit mining is often used for extracting “hard rocks” such as metal ores like gold, silver, copper, iron, among others. The process begins by excavating large surface areas of the ground to expose and mine the metal ore beneath. Specifically, holes are drilled into the ground using explosives, and hard rock material is then removed layer-by-layer through controlled demolitions.

8 Information on the timeline of mining exploration and exploitation activities was provided by the Mexican Geological Service (SGM) through a public information request system.

9 For legal documentation of *ejidos* and mining concessions, see article 27 of the mining law: http://www.diputados.gob.mx/LeyesBiblio/pdf/151_110814.pdf.

10 This study follows the theoretical framework of a non-unitary rather than unitary household bargaining model. A majority of empirical studies find little evidence in support of the unitary household model framework, which has been criticized for its over-simplicity and lack of applicability to real world data (Lundberg and Pollak, 1994; Browning et al. 2006; Bobonis 2009; Cherchye et al. 2012).

11 In this study, the *MxFLS* sample excludes women who got divorced (and who may or may not have changed partners). The same women (and partners) are therefore followed over time across all three survey waves to attenuate concerns relating to possible endogenous marriage formation.

12 Data and documentation are available at http://www.ennvih-mxfls.org/. An example of the household decision making questionnaire can be found in Fig. 6 of the appendix.

13 Specifically, I assign a value of 3 if a woman responded that she was the sole decision-maker over that category of goods and services, 2 if the decision was made jointly with her husband, and 1 if her husband was the sole decision-maker of that category of goods and services. Each decision-making question is then normalized with a range of 0 to 1. Using this information, I then construct an average decision-making power index over all twelve decisions. A one standard deviation change in the average DMP index is therefore equivalent to a unit change in the average DMP index. The percentage change in the outcome variable is subsequently calculated by dividing the DMP coefficient by the mean standard deviation of the outcome variable.

14 Reporting biases may arise from the ‘Hawthorne effect’, a psychological term referring to the alteration of one’s behavior or survey response due to the cognizance of being observed (Landsberger, 1957).

15 Since exploitation activities last on average between 20-30 years, I subtract 20-30 years from the time market speculation about the impending global financial crisis began in 2003, and thus use historical mining concessions from the 1973-1983 time period.

16 For legal documentation, see articles 15 and 19 of the mining law: http://www.diputados.gob.mx/LeyesBiblio/pdf/151_110814.pdf.

17 The Federal District of Mexico and Quintana Roo are not known to be ‘mining’ states in general. Data on the production of gold and silver is available on the state level, but only specifically for states that are major producers of the two metals. States that produce relatively less of these minerals (such as the Federal District of Mexico and Quintana Roo) are grouped together under ‘others’ in the data. It is therefore not possible to know the exact amount of gold and silver produced in the two states, in addition to the lack of historical mining concessions data which is used as a proxy for endowment in this study.

18 It is possible that the *ENDIREH* did not capture a particularly vulnerable age group of those below 15, since marriage as young as the age of 12 was legal in some states in Mexico during the sample period. Nonetheless, the average proportion of girls married below 15 was approximately only 3.3% out of all women who were married across the three *ENDIREH* survey rounds (INEGI, marriage statistics).

19 Transport network quality and community quality are indices calculated using standard principal component analysis. They include various transport network questions such as the kind of materials that the roads, streets or highways are made of, if cars were able to pass through the community in the last two years, inter alia, and community quality questions like flooding problems, air quality, the sufficiency of public light systems, among others.

20 Because homicides are least subject to failure in reporting and are the most consistent with multiple studies using drug-related crimes, they remain the best indicator for violent living conditions despite the availability of data on other types of crimes related to the drug war (Ellsberg et al. 2001; Brown and Velásquez 2017).

21 The *ENDIREH* contains a total of thirteen decision-making questions in the 2003 and 2011 surveys, though the 2006 survey only contains eleven questions. For consistency, I exclude the two questions that were missing from the 2006 survey, and subsequently select questions that are similar to the ones asked in the *MxFLS*. This results in a remainder of six suitable decision-making questions that are then used to construct the average decision-making power index. For example, questions like ‘*who makes decisions about your participation in social or political activitie*s*?*’ are excluded as they are not asked in the *MxFLS* data set.

22 Output is not listed, but available upon request.

23 For a detailed breakdown of the specific IPV questions and categorization, see Bobonis et al.’s (2013) online appendix: https://www.aeaweb.org/articles?id=10.1257/pol.5.1.179. In 2003, only twenty-nine questions on IPV were asked compared to the 2006 and 2011 survey rounds which had thirty IPV questions. I therefore only include the twenty-nine IPV questions from 2003 across all survey years to ensure consistency in the IPV measures.

24 According to Cameron et al. (2008), standard errors of the main IPV estimates are likely to be underestimated due to the small number of clusters (30 states), which is below the recommended benchmark of 40. As a robustness check, I therefore present wild-cluster bootstrapped standard errors in Table 12 of the appendix, which show that standard errors increase slightly as expected, and estimates remain statistically significant.

25 Women are considered employed if they had worked at least one hour in the past week. According to the INEGI’s definition, women living in localities with less than 2,500 inhabitants are classified as rural. Lastly, educated women are defined as those who have completed at least junior high school (‘secundaria’), which is Mexico’s basic compulsory education requirement.
